# Supplementary material for: Content-rich biological network constructed by mining PubMed abstracts
Source: BMC Bioinformatics. 2004 Oct 8;5:147. doi: 10.1186/1471-2105-5-147 (PMC528731; doi:10.1186/1471-2105-5-147)
Supplement: Additional File 2 — The original results of the above study (non-essential files are deleted to keep the file size under the limit set by BMC bioinformatics). [file 1471-2105-5-147-S2.bz2 › chilibotAdditionalFile2/dip05/660ID2118521E11051/html/CBBL_CBBS.html]

 


 **CBBL** and **CBBS** 
  
Found 12 abstracts in PubMed, retrieved 05.  
 

 What does Google say? 
 PDF only 
| .edu only 

---

**Interactive relationship** (e.g. stimulation, inhibition, etc)

**Neutral relationship**- The deduced gene products,  **CbbL**  and  **CbbS** , had 93 and 87% identity with Thiobacillus intermedius  **CbbL**  and Nitrobacter winogradskyi  **CbbS** , respectively.  Ref: 12005060 Biosci Biotechnol Biochem, 2002

**Non-interactive relationship** (e.g. studied together, co-existance, homology, etc.)

- Analysis of RNA showed that  **cbbL**  and  **cbbS**  are cotranscribed on a message approximately 00 nucleotides in size.  Ref: 9696760 J Bacteriol, 1998
- The deduced primary sequence  **cbbL**  and  **cbbS**  of form I ribulose bisphosphate carboxylase oxygenase rubisco from Bradyrhizobium japonicum places this enzyme within the Type IC subgroup of red like rubisco enzymes.  Ref: 9882445 Arch Biochem Biophys, 1999
- Genes encoding the large subunit  **cbbL** , small subunit  **cbbS**  and putative regulatory gene cbbQ were shown to be located on one cluster.  Ref: 11889481 Arch Microbiol, 2002
- Expression of  **cbbL**  and  **cbbS**  in Escherichia coli led to the detection of RubisCO activity in the presence of 0.1 mM isopropyl beta D thiogalactopyranoside IPTG.  Ref: 12005060 Biosci Biotechnol Biochem, 2002
- Isolation and characterization of  **cbbL**  and  **cbbS**  genes encoding form I ribulose bisphosphate carboxylase oxygenase large and small subunits in Nitrosomonas sp.  Ref: 12005060 Biosci Biotechnol Biochem, 2002
- In the present study,  **cbbL**  and  **cbbS**  were cloned and sequenced.  Ref: 9696760 J Bacteriol, 1998
- Four genes encoding carboxysome shell peptides csoS1A, csoS1B, csoS1C, csoS2, the genes encoding the large and small subunits of RuBisCO  **cbbL** ,  **cbbS** , and three unidentified ORFs constitute an operon in Thiobacillus neapolitanus.  Ref: 10816046 Arch Microbiol, 2000
- the genes for the enzyme,  **cbbL**  and  **cbbS** , are part of a putative carboxysome operon.  Ref: 9696760 J Bacteriol, 1998
- The  **cbbL**  and  **cbbS**  genes encoding form I ribulose bisphosphate carboxylase oxygenase RubisCO large and small subunits in the ammonia oxidizing bacterium Nitrosomonas sp.  Ref: 12005060 Biosci Biotechnol Biochem, 2002
